# Financial literacy and inclusion for rural agrarian change and sustainable livelihood in the Eastern Cape, South Africa

**DOI:** 10.1016/j.heliyon.2023.e16330

**Published:** 2023-05-19

**Authors:** J.O. Akande, Y.S. Hosu, H. Kabiti, S. Ndhleve, R. Garidzirai

**Affiliations:** a Department of Accounting, Walter Sisulu University, Mthatha, South Africa; b Department of Economics and Business, Walter Sisulu University, Mthatha, South Africa; c Risk and Vulnerability Science Centre, Walter Sisulu University, Mthatha, South Africa; d Department of Management, Walter Sisulu University, Butterworth, South Africa

**Keywords:** Financial literacy, Financial inclusion, Sustainable livelihood, Rural farmers, Structural equation modelling

## Abstract

Finance and growth underpin the debate on the implication of financial literacy and financial inclusion for development among scholars. While financial inclusion and financial literacy are positioned as enablers of sustainable development, literature has failed to link the two with sustainable development, especially for rural agrarian dwellers in dire need of financial development attributes. Therefore, this study applied variance-based structural equation modelling to examine the implication of financial literacy and financial inclusion for the sustainable livelihood of rural agrarian settlers in the Eastern Cape province of South Africa. Global scales were adapted and validated before administering them to 283 farmers and subsistence businesses in the region. The results revealed that financial literacy positively and significantly influences financial inclusion (β = 0.452) and the attainment of sustainable livelihood (β = 0.444). Financial inclusion partially mediates the influence of financial literacy on sustainable livelihood (β = 0.253), as it directly explains the province's sustainable livelihood (β = 0.114). The study's implication informs structured intervention for improving financial literacy to assist the target group with access to requisite funding, which could support their economic activities for a decent living.

## 1 Introduction

Financial inclusion and financial literacy have emerged as priorities for policymakers towards poverty alleviation in several developing countries. Financial inclusion is antithetical to financial exclusion, which has perennially conditioned the poor to a life of perpetual poverty [1]. describe financial exclusion as a situation in which people do not have access to mainstream financial products and services such as bank accounts, credit cards and insurance policies, particularly home insurance and education loans. The effects of financial exclusion can include exclusion from other mainstream services, such as pension or saving schemes. They can also lead to debt and/or being cut off from essential utilities. Several authors have aimed to define financial inclusion. While many authors see [[2], [3], [4], [5]] agree on the commonality of the definition of financial inclusion as financial infrastructure and access to mainstream financial products and services as the necessary bridge to prosperity, the discussions on financial inclusion have seldom discussed the pathway of financial literacy [2,4,5]. Financial literacy of the poor and the downtrodden will determine whether the financial products or services accessed by the vulnerable are merely for consumption or utilised for creating a sustainable livelihood. It is pertinent that the conversation about financial development proceed beyond the various aspects of financial infrastructure, legal background and the roles the demand side plays. It seems plausible that functioning financial markets require good infrastructure and informed customers, i.e. customers with a higher degree of financial literacy.

Financial literacy is challenging to define, considering its multidimensional angles in a complex economy [6]. However, this article focuses on its linkages to the vulnerable and sustainable livelihood systems. The terms ‘financial literacy’, ‘financial knowledge’ and ‘financial education’ are often used interchangeably in literature. [7], in their Presidential Advisory Council on Financial Literacy (PACFL) report, define financial literacy as the ability to use knowledge and skills to manage financial resources effectively for a lifetime of financial well-being. Other conceptual definitions continue to be used. However, it is unclear how widely the PACFL definition is accepted. One of the striking observations made from the literature is that financial literacy has been variably defined as (a) a specific form of knowledge, (b) the ability or skills to apply that knowledge, (c) perceived knowledge, (d) good financial behaviour and even (e) financial experiences.

There has been documentation of some successes of inclusive financial systems and their impact on sustainable development, especially in some parts of Asia [3]. [8] state that lack of financial inclusion is still a far-reaching problem in the bid for sustainable development. The Findex data for 2014 show that 2 billion adults are unbanked; this number fell to 1.7 billion in 2017, still representing almost 40% of adults worldwide. In sub-Saharan Africa, relative to other developed countries, the mature populace with accounts or borrowings from formal financial institutions continues to decline [9]. Heterogeneously, only 7% of the working-age citizens in Burundi, Guinea and Niger have a bank account, relative to 82%, 75% and 70% in Mauritius, Kenya and South Africa, respectively [10]. Financial inclusion, measured as access to and use of financial services, is an important goal of economic and, in particular, financial development; accordingly, it has been argued to be an important policy tool that can help to achieve the Sustainable Development Goals [11]. Improved financial inclusion can decrease rural poverty [12] and increase employment [13], expenditures [14] and savings [15]. Hence, better financial inclusion can have welfare effects that extend beyond benefits in the financial realm to the real economy. Financial inclusion and literacy play an important role in ensuring the financial health and stability of individuals, families, enterprises and national economies. Therefore, country studies on financial inclusion focus on the supply side of financial markets [16]. suggest that robust financial inclusion of the hitherto inadvertently financially excluded and the poor segments of society would increase economic activities, employment, consumption, government expenditure, economic growth and sustainable growth development.

The Eastern Cape province of South Africa was one of the homelands during apartheid with every manifestation of segregation and deprivation and loss of livelihood, where Black people depended mainly on the White economy [17]. [18] describe fiscal and intergovernmental asymmetry that existed among the local governments where the homelands were located, where Black people survived on subsidies without meaningful and sustainable livelihood activities. The manifestation was a high number of poor rural populations that are predominantly rural agrarian and non-agrarian, which is argued to be consistent with the level of financial literacy and the nature of financial services and products in terms of financial inclusion [19]. [19] documented the province as the least financial literate province in South Africa, with 43.1% financial literacy score. Financial products such as the number of automated teller machines (ATMs), bank branches and account holdings rank among the lowest among the provinces in South Africa [20], owing to the low-income status of the province [21]. The Eastern Cape is the poorest province in South Africa, with a poverty intensity of 43.3% [22], thereby making the issues of sustainable livelihood a grievous concern and study imperative.

The post-apartheid government of South Africa have developed several policies and interventions especially relating to developing agricultural capabilities to improve the lives of people living in the Eastern Cape, but these have not yielded tangible report [23]. [24] opine that historical processes, national policies and local dynamics have contributed to this situation. However [25], identified lack of budgeting, cost analysis and financial access as having contributed to the success or otherwise of farming households in the Eastern Cape. Yet, no study has directly examined the influence of financial literacy and financial inclusion and their directional impact on livelihood activities in the Eastern Cape. Hence, we investigated the implication of financial literacy and financial inclusion for the sustainable development of rural small farmers in the Eastern Cape.

**Fig. 1:**
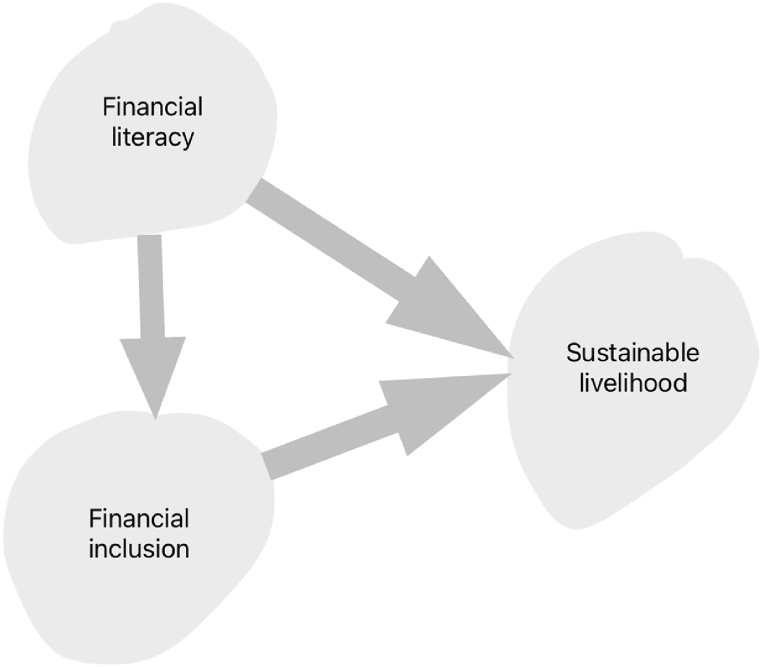
The study's conceptual framework.

This study extended literature in two unique ways. Firstly, it measured the combined implications of financial literacy and financial inclusion for sustainable livelihood represented in the conceptual framework in Fig. 1, showing, theoretically, the direct and indirect relationship of the phenomena to sustainable livelihood. Secondly, the relationships were explored for rural agrarian communities in South Africa. Higher-order structural equation modelling was implemented, and the findings revealed that financial inclusion and financial literacy directly impact sustainable livelihood. Furthermore, financial inclusion partially mediates the impact of financial literacy on sustainable livelihood. The study has implications for policies on improving poverty in the province in particular and in South Africa in general.

Section 2 of the article presents the literature review and hypothesis development, Section 3 discusses the methodology applied, Section 4 the results and discussions, while sections 5, 6 contains the summary, conclusion and policy implications.

## 2 Literature and hypotheses development

The importance of financial literacy and financial inclusion for poverty reduction and progress towards sustainable development cannot be disputed. In fact, in emphasising the role of financial literacy and financial inclusion in sustainable development, literature documents that economic growth does not portend individuals’ prosperity due to inequalities embedded in country systems [26]. [27] identified the importance of financial literacy in reducing inequalities and stresses the need for financial education for a country to be financially inclusive. These relationships are underscored by capabilities and human capital development theories that explain the importance of literacy for the potential to partake in financial systems, including the implication of literacy for development.

### 2.1 Financial literacy and financial inclusion

Financial literacy has a profound influence on financial inclusion in literature. According to Ref. [28], beneficiaries of financial training have greater chances of owning a bank account, including getting financial assistance, compared to non-beneficiaries. The authors posit that financially literate people have a better opportunity to be financially included [29]. found consistencies with the preceding arguments in that financial access and capabilities are facilitated by financial education that aids consumers’ return. An earlier study by Ref. [30] documented the relevance of financial literacy for financial inclusion among people, whether poor or rich. Not much literature has considered micro, small and medium enterprises and small farmers supporting the role of financial literacy in improving financial inclusion [2,31]. However, despite the potential of financial literacy in engendering financial inclusion [2], further noted the high prevalence of financial exclusion of small and marginal farmers and some social groups regarding access to credit from formal institutions.

Cross-country evidence also indicates the role of financial literacy in financial inclusion. In Uganda [32], found that financial literacy has a direct positive influence on financial inclusion [33]. studied the impact of income and financial literacy on financial inclusion in Nigeria. They found that literacy significantly influences savings patterns with formal and informal financial institutions. In China [34], revealed that financial literacy has a significant positive relationship with financial inclusion. The study mediated the relationship with digital financial product usage and found that the promotion of digital financial product usage and the achievement of the goal of advancing financial inclusion in China hinged on improving residents' financial literacy and popularising the use of the Internet. Similar results supporting the positive and significant impact of financial literacy on financial inclusion were reported across countries see Refs. [8,30], including Indonesia [31,35], Sri Lanka [27], Ghana [28] and Laos [36]. Evidence from South Africa by Ref. [37] indicates that financial literacy influences the use of financial products in the country. Therefore, the study's hypothesis that.

H11 Financial literacy positively influences the financial inclusion of Small Farmers and Small Businesses (SFSB) in the Eastern Cape, South Africa.

### 2.2 Financial inclusion and sustainable livelihood

A deluge of literature has explored the implication of financial inclusion for poverty alleviation. The consensus in the literature is that financial inclusion enhances the growth of economies, reduces poverty and brings about sustainable economic development. According to Ref. [38], accessing banking services enhances poverty alleviation beyond simply providing people with livelihood projects. An earlier study by Ref. [39] documented financial inclusion as a vehicle of development and a strategy for poverty alleviation. The author argues that increasing financial inclusion efforts provides an opportunity to tackle poverty and promote inclusive development [40]. explored financial inclusion levels and their potential impact on inclusive growth. Using relevant poverty reduction indices and indicators, the author concluded that the major setback to poverty reduction and economic development is non-accessibility to financial services. The conclusion was based on the findings that the depth of financial services remained low. Financial inclusion decreases the likelihood of poverty. According to the evidence provided by Ref. [28], financial inclusion is associated with a decline in households’ likelihood of being poor, including preventing exposure to future poverty. Furthermore, the authors found that financial inclusion reduces rural vulnerability to poverty more than in urban areas [26]. aver that sustainable development and finding a solution to the deep-rooted problem of poverty go beyond economic growth, as financial inclusion is required to play a role in the distribution of wealth and prosperity.

[41] argue that financial inclusion could help reduce the vulnerabilities of poor households and rural communities and improve economic resilience. The authors conclude that financial inclusion would bridge and enhance the achievement of sustainable development. Access to bank accounts as a measure of financial inclusion for women portends positive implications for inclusive economic growth [42]. investigated the effect of female financial inclusion in 91 emerging and non-emerging countries and found that greater financial inclusion of women in terms of usage and owning bank accounts, among other things, enhances the development of economies.

Although there is a plethora of literature on the impact of financial literacy on financial inclusion as well as financial inclusion on poverty reduction for sustainable development, not much has been done to explore the direct effects of financial literacy on sustainable livelihood. Country-level studies lend credence to these findings. Global studies from India, Kenya, Egypt, the Middle East and North Africa, Nigeria and Ghana, among other countries, are all rooted in the need for financial inclusion for economic emancipation see Refs. [28,40,43]. A study on southern Africa by Ref. [39] also supports the role of financial inclusion in lifting people out of poverty. Furthermore, Fanta et al. [44] link social grants with financial inclusion and argue that the digitisation of social grants provides financial inclusion for those in that safety net in the South African space. In addition, there is scarce literature exploring the role of financial literacy and financial inclusion in the sustainable livelihood of SFSB in South Africa. Thus, the study pursues that hypothesis that.

H21 Financial inclusion positively influences the sustainable livelihood of SFSB in the Eastern Cape, South Africa.

### 2.3 Mediation and literature gap

South Africa faces numerous challenges in terms of financial inclusion despite the country having advanced and complex financial systems [45]. These complex financial systems act as barriers to entry for people with low literacy levels and those residing in rural spaces. This exclusion can be seen as part of the manifestation of the inequalities in South Africa. As such, innovative and context-specific approaches are prerequisites to addressing the financial exclusion challenge [45]. Given the challenge of financial inclusion, it is essential to invest in financial literacy to empower people living in rural areas. Financial literacy ensures that informed financial decisions are taken at individual and household levels due to understanding money and managed practices [46]. In addition, financial literacy increases life skills and bargaining power through knowledge of current pricing, financial systems and options. This all contributes to the strengthening of rural livelihood towards sustainability. Despite the direct impact these related financial literacy features have on the sustainable livelihood of rural lives, their role in sustainable livelihood have, at best, been remote and indirect through financial inclusion [47]. Therefore, this study hypotheses the direct influence of financial literacy on sustainable livelihood and the mediating role of financial inclusion in the relationship between financial literacy and sustainable livelihood as follows.

H31 Financial literacy positively influences the sustainable livelihood of SFSB in the Eastern Cape, South Africa.

H41 Financial mediation positively mediates the influence of financial literacy on the sustainable livelihood of SFSB in the Eastern Cape, South Africa.

## 3 Materials and methods

A cross-sectional research design was considered appropriate for this study. The advantage of a well-designed cross-sectional survey is the potential to support robust analysis [48]. This assists in analysing the implications of financial literacy and financial inclusion for the sustainable livelihood of SFSB in the Eastern Cape.

The study was conducted using data from the agrarian areas of grassland (Mthatha), savanna (East London) and Karoo (Queenstown), constituting the three agricultural areas within the province. The targeted samples are located in the rural settlements of the areas where they conduct their subsistence businesses and farming. All the participants provided information based on their holding of either small farming activities or subsistent businesses.

Amid the several but similar global scales that exist for measuring financial literacy, financial inclusion and sustainable livelihood in literature, this study adopted the scales used in Refs. [[49], [50], [51], [52], [53]]. Notwithstanding the adoption of the scales, the instruments were piloted, and the process involved removing items that did not apply to this study [54]. These formed the basis on which participants were scored on latent factors such as financial skills and financial knowledge for financial literacy; access, quality and welfare for financial inclusion; and social and financial capital for sustainable livelihood.

The survey consisted of two parts. Given the emphasis on SFSB, the first part involved a business nature profile that gauged the size and scope of business, dealing with farm size, enterprise type, business scale and revenue. The second part dealt with the three key concepts of the study. Two latent measures, namely financial skills and knowledge, emerged for financial literacy, each coded with three questions and each considering the standard measures available in the literature. A similar scenario played out for financial inclusion and sustainable livelihood. The former had three latent measures, namely access, quality and welfare; the latter two coded with four questions, with access having three questions. In the latter case, two latent factors, namely financial capital and social capital, arose from the list of factors traditionally considered in the literature and were coded with four questions each. The questions were accordingly ranked on a five-point scale (strongly disagree, disagree, neutral, agree and strongly agree), ranging from 1 to 5 in that order. Participants were to indicate their responses to the various questions posed on the scale.

A questionnaire instrument was utilised for the study. Ethical clearance was obtained from the university for the instrument, which included a consent form that ensured that the consent of the participants was obtained prior to data collection. The questionnaires were hand-distributed by appointed enumerators to the participants under supervision at the three targeted farming locations within the province. The SFSB were sampled using a combination of simple random probability and snowball sampling techniques, provided they met the criteria qualifying them to be SFSB. These farmers are those that depend mainly on rainfall for their production, cultivate less than five acres of land and have limited access to the market. This study considered subsistence businesses as small businesses that only sell for survival, unlikely to grow to the level of creating jobs. The simple random probability sampling method ensured that each member of the targeted farming and business population had an equal chance of being sampled, keeping the process less biased with the potential for a representative sample [55,56]. The snowball approach enabled the known contacts to recommend other participants who partook in the study, which became particularly handy, as the population of the study is unknown [57].

We were unable to obtain the study population. Hence, we relied on some studies to consider an appropriate sample size as far as working within partial least squares structural equation modelling (PLS-SEM), the estimating techniques used in this study [58], measuring of relationships [59] and studies with unknown population [57], are concerned. In total, 283 participants completed the questionnaires which are considered appropriate for the study analysis given the foregoing. Of the total data collected, 83 participants were from the grasslands and 100 each from the savanna and the Karoo.

The disjointed two-stage reflective-reflective high-order model was the approach used for this study, given the expectation of high correlation among the lower- and the higher-order constructs with unequal latent factor items [60]. This procedure allowed all the lower-order constructs for the latent factors of financial literacy, financial inclusion and sustainable livelihood, to be modelled and latent variables scores obtained to create and measure the higher-order constructs. Three direct effects and one indirect effect hypothesised were modelled through the disjointed two-stage reflective-reflective high-order approach to implement the study's objective. The application of this approach requires the creation and estimation of the model connecting all the lower-order constructs. The lower-order construct score became input for the higher-order constructs used in the study model to evaluate the hypotheses under consideration. The analysis was implemented in a variance-based structural equation model that is conventionally a domain of the PLS-SEM. The choice of PLS-SEM was informed by its flexibility to estimate complex relational models having many constructs, indicator variables and structural paths in a study this nature that has higher-order constructs without imposing distributional assumptions on the data see [61].

A couple of diagnoses have been adopted in literature to achieve the measurement models that establish the reliability and validity of the constructs [62,63] implemented in this study. These include checking the items' factor loading for the reliability of the indicators, composite reliability (CR) and the Cronbach's alpha for scale reliability, the average variance extracted (AVE) for convergent validity and the Fornell-Larcker criterion cross-loading as well as the heterotrait-monotrait ratio (HTMT) for discriminant validity.

## 4 Results and discussions

### 4.1 Demographic characteristics

This study investigated the implications of financial literacy and financial inclusion and their combined effects on the sustainable livelihood of SFSB in the Eastern Cape. Data were obtained from the participants across the agrarian divides in the province. The profile of the participants indicated that of the 283 participants, 210 were women, 160 were categorised as above youthful age, 192 had up to Grade 12 education with others having tertiary education, and 220 obtained their financing from commercial banks. In general, based on the criteria indicated in Section 3, all the participants owned either a small farm or a subsistent business in a ratio of 40:60. The major highlight of the participants’ profile statistics suggested some level of education, which could drive financial literacy, even at the grassroots. Moreover, the number of banking participants indicated a considerable level of financial inclusion, suggesting a correlation between the level of education and financial inclusion.

### 4.2 First-order analysis

The main aim of the first-order analysis is ensuring that the scores generated for the second-order analysis are valid, reliable and fit for purpose; hence the focus is not on the path analysis. Table 1 presents the results of the factor loadings, the data description and the measure of the multicollinearity model. The factor loading lies between 0.715≤fl ≤ 0.944 across the indicators above the 0.60 recommended as an acceptable quality of correlation among items in a construct [64]. Statistically, the mean response values range between 3.049≤x̄≤4.127 above the scale average with σ ≥ 0.50 attesting to a well-engaged instrument. For the lower-order construct structural model, the variance inflation factor (VIF) statistic is employed to evaluate multicollinearity in the indicators [65]. Following [66], multicollinearity is not a concern if VIF <5, as is the case in the results presented in Table 1.

**Table 1:** Factor loadings, descriptive statistics and multicollinearity.

	Indicators	Loadings	Mean	SD	VIF
Financial skills	fsl2	0.906	4.088	0.821	2.930
	fsl3	0.925	4.099	0.896	3.431
	fsl4	0.922	4.042	0.885	2.753
Access	acc1	0.882	3.763	1.167	1.426
	acc3	0.763	3.943	0.997	2.501
	acc4	0.813	3.933	0.980	3.102
	acc5	0.803	3.996	0.975	2.658
Financial knowledge	fkl2	0.801	4.127	0.852	1.342
	fkl5	0.882	3.378	1.273	2.819
	fkl6	0.859	3.216	1.277	2.722
Financial capital	fnc1	0.723	3.618	1.330	1.377
	fnc2	0.845	3.406	1.343	1.765
	fnc3	0.808	3.018	1.251	2.344
	fnc4	0.785	3.049	1.310	2.199
Quality	qlt1	0.944	3.654	1.171	4.713
	qlt2	0.934	3.569	1.293	4.328
	qlt4	0.927	3.664	1.095	2.986
Social capital	scc1	0.715	3.954	1.122	1.250
	scc2	0.889	3.671	1.178	3.145
	scc3	0.788	3.664	1.164	3.004
	scc4	0.796	3.640	1.229	2.533
Welfare	wel1	0.839	3.304	1.156	2.561
	wel2	0.940	3.378	1.129	4.782
	wel3	0.944	3.254	1.203	4.825
	wel4	0.895	3.378	1.181	3.120

The scale reliability and convergent validity results are presented in Table 2, namely Cronbach's alpha and CR for reliability and AVE for convergent validity. The Cronbach's alpha results lie within 0.802≤α ≤ 0.929, while CR has values within 0.870≤CR ≤ 954. Although [67] have some issues about CR values being >0.95, the two measures were still considered acceptable reliability thresholds for this study [see 66]. Similarly, the AVE range within 0.626≤AVE≤0.875 above the threshold of 0.50 for all the lower-order constructs suggests the internal consistency of the items within each construct [66].

**Table 2:** Scale reliability and convergence validity analysis.

	Cronbach's alpha	rho_A	CR	AVE
Access	0.864	1.165	0.889	0.666
Financial capital	0.802	0.814	0.870	0.626
Financial knowledge	0.806	0.811	0.885	0.719
Financial skills	0.907	0.921	0.941	0.842
Quality	0.929	0.938	0.954	0.875
Social capital	0.820	0.841	0.876	0.639
Welfare	0.927	0.956	0.948	0.821

Next is the measure of discriminant validity based on the Fornell-Larcker criterion [65] in Table 3; cross-loading and the HTMT are both contained in Appendix 1, tables A and B, respectively. For cross-loading, the rule of thumb is for the items within a construct to load higher on their intended construct than any other construct. Thereafter, each construct AVE has to be higher than the squared correlation between any of the different constructs. The last and preferred measure is the HTMT ratio [67]. It is expected to be less than one to ascertain that each item within a construct is more highly correlated with its parent construct than any other construct. It therefore compares the ratio between trait correlations to the ratio of within-trait correlations to estimate the actual correlation between perfectly measured variables [68,69]. Previous debates consider HTMT of ≤0.85 thresholds as appropriate, but a more liberal threshold favours ≤0.90 see Refs. [69,70]. All results of these measures confirmed the discriminant validity of this study.

**Table 3:** Discriminant validity (Fornell-Larcker criterion).

	Latent factors	1	2	3	4	5	6	7
1	Access	**0.816**						
2	Financial capital	0.333	**0.791**					
3	Financial knowledge	0.328	0.597	**0.848**				
4	Financial skills	0.309	0.336	0.591	**0.918**			
5	Quality	0.616	0.299	0.386	0.348	**0.935**		
6	Social capital	0.315	0.540	0.416	0.342	0.425	**0.800**	
7	Welfare	0.548	0.381	0.403	0.316	0.752	0.333	**0.906**

### 4.3 Higher-order analysis

Having confirmed the reliability and validity of the lower-order constructs, the scores of the constructs were inputted into the higher-order construct in a reflective model to generate the convergent and discriminant validity, presented in Table 4. The higher-order construct measurement model assessment was also validated such that each of the constructs of financial literacy, financial inclusion and sustainable livelihood were assessed for reliability, convergent validity and discriminant validity [60]. Similar to the lower-order constructs, the same criteria and thresholds applied to these measures. To that extent, the reliability and validity of these constructs were confirmed for this study. However, only the results of the Fornell-Larcker criterion are presented for discriminant validity and were considered appropriate on the strength of the outcomes of the lower-order construct measures.

**Table 4:** High-order reliability and validity.

	Latent factors	Cronbach's alpha	rho_A	CR	AVE	1	2	3
1	Financial inclusion	0.841	0.847	0.905	0.760	**0.872**		
2	Financial literacy	0.743	0.791	0.884	0.792	0.452	**0.890**	
3	Sustainable livelihood	0.701	0.711	0.869	0.769	0.454	0.559	**0.877**

### 4.4 Structural model

**Fig. 2:**
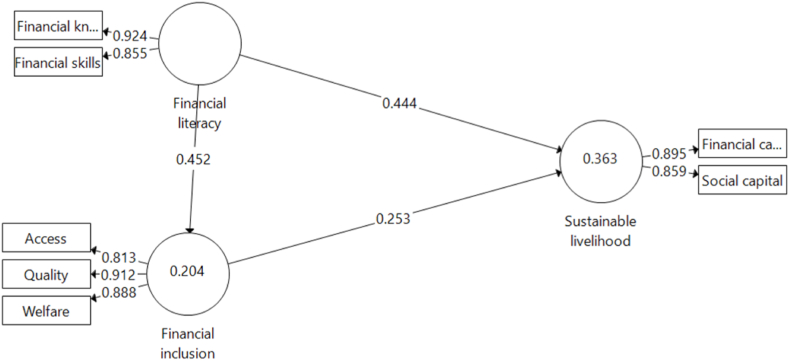
Second-order structural model.

Following the validation of measurements and the structural models for the lower-order constructs and higher-order constructs, it is certified that the models are fit for purpose. The hypotheses proposed for this study were assessed to ascertain the evaluated relationships. The structural model results for the hypothesis under consideration are presented in Fig. 2 for path analysis, Appendix 2 for the bootstrapping model containing the *t*-test and Table 5 for the regression model summary. The lower-order construct structural model is also presented in Appendix 3 for reference. As seen in Fig. 2 and Table 5, three direct paths and one indirect path were tested, corresponding to this study's various hypotheses.

**Table 5:** Path regression model.

Hypothesis	Direct effects	β	SD	T-stat	P	Decision
**H1**	Financial literacy - > Financial inclusion	0.452	0.068	6.620	0.000	Supported
**H2**	Financial literacy - > Sustainable livelihood	0.444	0.045	9.781	0.000	Supported
**H3**	Financial inclusion - > Sustainable livelihood	0.253	0.057	4.419	0.000	Supported
	**Indirect effect**					
**H4**	Financial literacy - > Financial inclusion - > Sustainable livelihood	0.114	0.034	3.365	0.001	Supported

The first hypothesis measured the path from financial literacy to financial inclusion, testing the alternate assertion that the former positively influences the latter for SFSB within the study contexts. The result showed that financial literacy positively and significantly affects financial inclusion. More specifically, a 1% increase in financial literacy can potentially increase financial inclusion by approximately 45.2%. This result is consistent with the hypothesis of this study and the *a priori* expectation that the more financially skilful and/or knowledgeable an individual becomes, the better that individual should be able to access quality financial products. This study participants’ education attainment seemed to corroborate this result in the region, which may have been influenced by their literacy rate and which in turn may have driven their financial awareness.

Although the lowest in South Africa [19], put the literacy score in the Eastern Cape at 43.1%. Theoretically, the results find consistency with the capability theory that envisages that individuals' potential to participate in financial systems is enhanced by their ability to develop their literacy on financial traits [71]. This study's findings also align with some empirical literature that has established a positive and significant relationship between financial literacy and financial inclusion [28]. [28] showed that people with financial training have greater chances of owning a bank account, including getting financial assistance, bolstered by this study's participants, indicating that over 70% of the participants banked with a formal financial institution [30]. explain the relevance of financial education access in less-developed economies as against the usage of financial products, which might have described why usage as a construct of financial inclusion was unsupported in the analysis of this study. The few pieces of literature that have considered this relationship between micro, small and medium enterprises and small farmers support the role of financial literacy in improving financial inclusion [31]. Although [2] laments the exclusion of small farmers, marginal farmers and some social groups in terms of access to credit from formal institutions, one wonders whether that can still be the case today, given the findings in this study. Our study is in sync with country-level studies such as those of [32] in Uganda [72], in Kenya [73], in Georgia [34], in China [28],in Ghana and [37]in South Africa. All these studies came to a similar conclusion about the direct positive impact of financial literacy on financial inclusion across various countries. It is important to note that usage as a factor of financial inclusion was not as significantly influenced in most of the studies as in this study, which [73] put succinctly to be a result of people's basic financial knowledge.

The second path of the model explored the relationship between financial literacy and the sustainable livelihood of the SFSB in the Eastern Cape. The result of the relationship showed a significant and positive 0.444 coefficient, implying that a 1% change in financial literacy could increase sustainable livelihood by 44.4%. This immense impact confirms our second hypothesis that financial literacy positively impacts the sustainable livelihood of SFSB in the Eastern Cape. This result is consistent with the human capital development theory [74]. likens livelihood to human, physical, natural, financial, and social capital that households engage in obtaining a living. Studies exploring how livelihood is sustained through financial literacy are scarce in the existing literature. Extant literature that has considered the relationship between financial literacy and sustainable livelihood has at best been implied, as the literature has been on poverty reduction instead of the specific activities in which the rural dwellers engaged for a living [27]. identified the importance of financial literacy in reducing inequalities and stresses the need for financial education for a country to be financially inclusive. Financial access is facilitated by financial education, translating into consumer return [29], therefore providing credence in empirical literature for this study's results that revealed a significantly positive relationship between financial literacy and sustainable livelihood. This study contributes to the empirical literature by testing the direct relationship between these phenomena, given that extant literature on the subject matter has been remote and primarily through financial inclusion [47]. The understanding is that financial literacy on its own is a means to using the financial product, which should cause poverty reduction and does not by itself has direct influence on the livelihood.

The third hypothesis, corresponding to the third path on the structural model in Fig. 2, tested a direct positive relationship between financial inclusion and sustainable livelihood of SFSB in the Eastern Cape. The result showed that financial inclusion has a direct, positive and significant relationship with sustainable livelihood, although with lesser explanatory power than financial literacy. According to the finding, this result confirmed the study hypothesis and aligned with expectations and empirical literature. It showed that a +1% change in financial inclusion results in a 25.3% increase in the sustainable livelihood of SFSB in the Eastern Cape. As the summary statistics and literature suggest, financing access, quality and welfare have increased over time. For instance Ref. [20], report an increase in the region's ATMs and borrowing rate. Although these statistics are not particularly targeted at the SFSB, they may not be unconnected with the positive influences of financial inclusion on sustainable livelihood witnessed in the region. In terms of empirical support [42], liken women's financial empowerment to economic development, which would indirectly emanate from a culmination of sustainable livelihood of the women. Literature conceded the role of financial inclusion in poverty reduction, economic growth and sustainable economic development [see 28, 43, among others]. This outcome provides credence to this study's result, as no specific literature relating financial inclusion to sustainable livelihood exists. [39], who conducted a southern African study, supports the role of financial inclusion in lifting people out of poverty. The researchers in this study expected financial inclusion to have a more direct impact on sustainable livelihood than financial literacy, but this was not the case, judging from the explanatory power of the relationship. The explanatory power of financial inclusion may not be unconnected with some of the challenges highlighted in literature within the region and in South Africa regarding high fees and affordability, banks' motives, mistrust and the regulatory environment, among other issues [21,45,75].

Finally, the last hypothesis tested the indirect relationship between financial literacy and sustainable livelihood through financial inclusion and found a positive and significant partial mediation with a 0.114 coefficient. While this conforms with the study's hypothesis and *a priori* expectation, it is also momentous in understanding the overall importance of financial literacy and financial inclusion in enhancing the livelihood of rural dwellers [47]. provided initial evidence, explaining that the role of financial literacy in terms of sustainable livelihood has at best been remote and indirect through financial inclusion. While this may be corroborated with this result, this study also found that financial literacy is even more critical in the direct relationship than being mediated by financial inclusion. This revelation brings to the fore the practicality of the cost of engendering financial inclusion for sustainable livelihood. At the same time, sustainable livelihood could have been directly achieved by enhancing financial literacy, which should guide policy direction.

The key findings from the foregoing revealed that financial literacy is essential for financial inclusion and sustainable livelihood. Contrary to the focus in literature, financial literacy has more power in driving sustainable livelihood than financial inclusion. Furthermore, financial literacy greatly influenced access, quality and welfare as financial inclusion constructs. Unexpectedly, financial literacy has a more direct influence on sustainable livelihood than financial inclusion, as opposed to the findings in conventional literature. Although financial literacy also indirectly influences sustainable livelihood through financial inclusion, it is not at the level expected to warrant such mediation. Increased efforts could enhance financial literacy to produce better output for improved livelihood sustainability. Suffice it to say that even though the findings of this study conform with extant literature in terms of the signs and direction of causality, the explanatory power differs, suggesting that different subsectors of an economy could react in different ways to these phenomena. These results have a couple of implications for policy for the sustainability of farming and small businesses in South Africa. The approach should be more directed at financial literacy, which enhances financial inclusion and could assist SFSB in creating wealth without having to solely depend on financial products from formal financial institutions, especially as usage was not fundamental in this study.

Moreover, increasing financial literacy beyond primary financial education becomes necessary, as that could catalyse understating of the different available financial products, such that the effect of financial inclusion on sustainable livelihood could be deepened. Beyond the short training and workshops usually organised, a more advanced certificate product may be recommended to be created for optimal usage.

## 5 Summary and conclusions

This study empirically investigated the implication of financial literacy and financial inclusion for sustainable development within the capabilities framework, human capital and finance and growth theories. Four hypotheses were tested, financial literacy and financial inclusion, financial literacy and sustainable livelihood, financial inclusion and sustainable livelihood, and the mediating role of financial inclusion in financial literacy and sustainable livelihood relationship. Survey questionnaires were deployed to collect data from the three agricultural areas of the Eastern Cape, namely the grasslands, savanna and Karoo. The disjointed two-stage reflective-reflective high-order PLS-SEM model was implemented to analyse the data collected from 283 participants. The study found evidence that financial literacy is essential in sustaining the livelihood of SFSB within the study area. Although financial inclusion is also relevant, the impact, according to the literature, is dampened by the product usage level arising from the individuals’ basic financial knowledge. Again, the study found that financial inclusion partially mediates financial literacy and sustainable livelihood and that the need to pursue such mediation left much to be desired, given the explanatory power of mediation.

The limitation of the study relates to the representative sample because of the difficulty in determining the sample population. Moreover, the project funding could not afford to keep enumerators in the field for long enough to collect as much data as desired. Future research needs to pay attention to more robust data collection. As this is a critical sector of the economy, deliberate efforts should be made to conduct these surveys regularly to be available for studies of this nature.

## 6 Policy implications

The results from the modelling and analysis are illustrative and offer vital insight into developing policies and interventions for sustainable livelihood development in the Eastern Cape. We therefore suggest the following policy interventions:

Firstly, the findings indicated that having formal education does not necessarily translate into having financial literacy skills for people to actively participate in sustainable livelihood activities. Policies need to be directed to build in financial literacy into curriculum in grade schools and also provide urgent intervention on specific financial literacy skills such as saving and how to save, how to budget, applying for loans and loan management, and how to invest. Such specific financial literacy will empower micro, small and medium enterprise owners and further remove barriers to mainstream financial products.

Secondly, given that financial inclusion both direct and mediating impact on sustainable livelihood system among the SFSB in Eastern Cape, a targeted policy intervention on financial inclusion is advised rather than general intervention that may be difficult to monitor.

Lastly, the majority of the business owners interviewed were women. This suggests that any policy intervention for financial literacy and inclusion must be gender-sensitive and must be pro-women.

## Funding

The project is funded by the Research and Innovation Directorate of Walter Sisulu University, Mthatha, South Africa (under the instrument of Small-scale Agribusiness and Rural Non-farm Enterprises Research Niche Area).

## Author contribution statement

All authors listed have significantly contributed to the development and the writing of this article.

## Data availability statement

The article literature utilised already available materials duly referenced and raw data collected are curated based on the funder's policy guided by applicable regulations in the jurisdiction.

## Declaration of competing interest

The authors declare that they have no known competing financial interests or personal relationships that could have appeared to influence the work reported in this paper.

## Figures and Tables

**Table A:** Discriminant validity (cross-loading)

Indicators	Access	Financial capital	Financial knowledge	Financial skills	Quality	Social capital	Welfare
FSL2	0.229	0.304	0.544	0.906	0.296	0.280	0.270
FSL3	0.242	0.286	0.546	0.925	0.304	0.284	0.287
FSL4	0.361	0.330	0.539	0.922	0.351	0.366	0.310
acc1	0.882	0.390	0.409	0.398	0.669	0.403	0.626
acc3	0.763	0.154	0.093	0.060	0.311	0.063	0.219
acc4	0.813	0.182	0.180	0.156	0.368	0.137	0.315
acc5	0.803	0.182	0.153	0.144	0.415	0.173	0.341
fkl2	0.317	0.608	0.801	0.512	0.340	0.335	0.416
fkl5	0.277	0.458	0.882	0.499	0.352	0.349	0.348
fkl6	0.224	0.420	0.859	0.483	0.276	0.375	0.232
fnc1	0.290	0.723	0.527	0.375	0.323	0.464	0.273
fnc2	0.268	0.845	0.555	0.328	0.222	0.491	0.361
fnc3	0.326	0.808	0.398	0.196	0.221	0.393	0.308
fnc4	0.148	0.785	0.365	0.110	0.165	0.325	0.244
qlt1	0.534	0.289	0.331	0.274	0.944	0.387	0.690
qlt2	0.520	0.224	0.307	0.291	0.934	0.400	0.643
qlt4	0.657	0.318	0.431	0.396	0.927	0.403	0.764
scc1	0.292	0.316	0.393	0.381	0.465	0.715	0.380
scc2	0.255	0.550	0.346	0.260	0.345	0.889	0.256
scc3	0.197	0.443	0.277	0.186	0.162	0.788	0.112
scc4	0.216	0.440	0.244	0.174	0.261	0.796	0.210
wel1	0.423	0.220	0.251	0.155	0.673	0.240	0.839
wel2	0.525	0.347	0.385	0.343	0.716	0.369	0.940
wel3	0.530	0.404	0.432	0.340	0.728	0.337	0.944
wel4	0.488	0.374	0.354	0.260	0.613	0.235	0.895

**Table B:** Discriminant validity (HTMT)

	Latent factors	1	2	3	4	5	6	7
1	Access							
2	Financial capital	0.319						
3	Financial knowledge	0.291	0.707					
4	Financial skills	0.257	0.371	0.686				
5	Quality	0.577	0.336	0.433	0.370			
6	Social capital	0.275	0.658	0.482	0.354	0.438		
7	Welfare	0.495	0.422	0.440	0.328	0.806	0.334	

## Appendices

### Appendix 1

Validity analysis.

### Appendix 2

First-order structural model.

### Appendix 3

Second-order bootstrapping model.
